# Development and validation of a screening questionnaire for early identification of pregnant women at risk for excessive gestational weight gain

**DOI:** 10.1186/s12884-023-05569-7

**Published:** 2023-04-13

**Authors:** Kristina Geyer, Roxana Raab, Julia Hoffmann, Hans Hauner

**Affiliations:** 1 Institute of Nutritional Medicine, Else Kröner-Fresenius-Centre for Nutritional Medicine, School of Medicine, Technical University of Munich, Georg-Brauchle-Ring 62, 80992 Munich, Germany; 2 European Foundation for the Care of Newborn Infants, Hofmannstrasse 7a, 81379 Munich, Germany

**Keywords:** Pregnancy, Excessive gestational weight gain, Risk score, Screening questionnaire, Prevention, Routine care, Obesity, GeliS

## Abstract

**Background:**

Excessive weight gain during pregnancy is associated with adverse health outcomes for mother and child. Intervention strategies to prevent excessive gestational weight gain (GWG) should consider women’s individual risk profile, however, no tool exists for identifying women at risk at an early stage. The aim of the present study was to develop and validate a screening questionnaire based on early risk factors for excessive GWG.

**Methods:**

The cohort from the German “Gesund leben in der Schwangerschaft”/ “healthy living in pregnancy” (GeliS) trial was used to derive a risk score predicting excessive GWG. Sociodemographics, anthropometrics, smoking behaviour and mental health status were collected before week 12^th^ of gestation. GWG was calculated using the last and the first weight measured during routine antenatal care. The data were randomly split into development and validation datasets with an 80:20 ratio. Using the development dataset, a multivariate logistic regression model with stepwise backward elimination was performed to identify salient risk factors associated with excessive GWG. The β coefficients of the variables were translated into a score. The risk score was validated by an internal cross-validation and externally with data from the FeLIPO study (GeliS pilot study). The area under the receiver operating characteristic curve (AUC ROC) was used to estimate the predictive power of the score.

**Results:**

1790 women were included in the analysis, of whom 45.6% showed excessive GWG. High pre-pregnancy body mass index, intermediate educational level, being born in a foreign country, primiparity, smoking, and signs of depressive disorder were associated with the risk of excessive GWG and included in the screening questionnaire. The developed score varied from 0–15 and divided the women´s risk for excessive GWG into low (0–5), moderate (6–10) and high (11–15). The cross-validation and the external validation yielded a moderate predictive power with an AUC of 0.709 and 0.738, respectively.

**Conclusions:**

Our screening questionnaire is a simple and valid tool to identify pregnant women at risk for excessive GWG at an early stage. It could be used in routine care to provide targeted primary prevention measures to women at particular risk to gain excessive gestational weight.

**Trial registration:**

NCT01958307, ClinicalTrials.gov, retrospectively registered 9 October 2013.

**Supplementary Information:**

The online version contains supplementary material available at 10.1186/s12884-023-05569-7.

## Background

Pregnancy can change a woman´s weight over her lifespan [1] through excessive gestational weight gain (GWG) and subsequent postpartum weight retention, thereby contributing to the development of overweight and obesity [2, 3]. Excessive GWG is known to be associated with unfavourable pregnancy and birth complications, such as gestational diabetes mellitus (GDM), preterm birth and caesarean delivery [4]. It further poses adverse risks to maternal and infant long-term health including the development of type 2 diabetes in mothers [5] and overweight and obesity in children [6]. In 2009, the US Institute of Medicine (now named National Academy of Medicine, NAM) [5] published evidence-based recommendations regarding an adequate GWG according to maternal body mass index (BMI) before pregnancy. Pregnant women who enter pregnancy with underweight, normal weight, overweight or obesity are recommended to gain weight between 12.5–18.0 kg, 11.5–16.0 kg, 7.0–11.5 kg and 5.0–9.0 kg respectively. Weight gain above the recommended ranges was defined as excessive GWG [5]. Along with the global rise of obesity [7], the prevalence of excessive GWG is increasing worldwide. According to a recent meta-analysis using individual participant data from over 218,000 pregnant women across 33 cohorts around the globe, almost 44% of women exceed the recommendations of adequate weight gain during pregnancy [8].

Multiple randomised controlled trials have aimed to avoid excessive GWG by lifestyle interventions. However, systematic reviews and meta-analyses summarising the findings of the individual studies showed only moderate to small reductions in GWG [9, 10], without improvements in most associated adverse pregnancy outcomes [4]. These results may indicate that intervention strategies should shift from a one-size fits all approach to a more targeted approach with potential adaptations according to one’s individual risk profile, eventually with more frequent intervention sessions [11]. Hence, early identification of a woman's individual risk profile for excessive GWG may provide an opportunity for offering personalised intervention strategies and avoiding unnecessary interventions.

Various factors that seem to influence the occurrence of excessive GWG have already been examined in multiple studies. A recently published systematic review and meta-analysis that included 70 studies with more than 3.3 million participants identified 58 different determinants of excessive GWG at the individual, family and social level [12]. The meta-analysis concluded that pre-pregnancy overweight and obesity, younger age (≤ 30 years), unemployment, being unmarried or divorced, primiparity and maternal smoking were associated with an elevated risk for excessive GWG [12]. According to findings from current literature there is still an inconclusive relationship between educational level, sociodemographic characteristics of pregnant women [12–14], parity [12, 15] or psychological features [12, 16, 17] and excessive GWG. Various studies reported that excessive GWG is associated with foreign nationality [18], migration background [19] or European ethnicity [20]. The studies mostly considered risk factors individually. The investigation of a combined risk model for excessive GWG, incorporating various potential risk factors, and thereon the development of a screening tool has not been carried out.

For elucidating the most important risk factors within one model, well-defined cohorts with extensive datasets are required. The German large-scaled, cluster-randomised, controlled GeliS (“Gesund leben in der Schwangerschaft”/“healthy living in pregnancy”) study originally aimed to prevent excessive GWG, however, no intervention effect was obtained [21]. The GeliS cohort, with its comprehensive dataset on maternal lifestyle and health outcomes, allows the assessment of various risk factors for excessive GWG. The aim of this analysis was to develop and to validate an easy-to-use and non-invasive screening questionnaire incorporating maternal anthropometric and sociodemographic factors, smoking behaviour as well as mental health status to identify pregnant women at risk for excessive GWG at an early stage.

## Research design and methods

This analysis was following the evidence-based guidelines for the “Transparent Reporting of a Multivariable Prediction Model for Individual Prognosis or Diagnosis: the TRIPOD statement” [22].

### The GeliS study

#### Study design and participants

The GeliS study is a large multicentre, cluster-randomised, controlled open intervention trial that was conducted within the routine health care system in five regions of Bavaria, South Germany. The sample size calculation of the GeliS trial was based on excessive GWG as primary endpoint and has been described elsewhere [23]. Between 2013 and 2015 pregnant women were recruited in 71 gynaecological and midwifery practices before the end of the 12^th^ week of gestation. Women were eligible for study participation if they were aged between 18 and 43 years, had a pre-pregnancy BMI between 18.5 kg/m^2^ and 40.0 kg/m^2^, a singleton pregnancy, sufficient German language skills, and provided written informed consent. Women with a complicated or multiple pregnancy or with severe illnesses were excluded as described elsewhere [23]. Women in the intervention group received a comprehensive lifestyle intervention programme that consisted of three structured face-to-face antenatal and one postpartum session alongside routine care visits. Women in the control group obtained routine prenatal care and leaflets with brief general information on a healthy lifestyle in pregnancy [23]. The study complied with the Declaration of Helsinki and local regulatory requirements and laws. The study protocol was approved by the ethics committee of the Faculty of Medicine of the Technical University of Munich and is registered in the ClinicalTrials.gov Registration System (NCT01958307).

#### Outcomes and data collection

The primary endpoint of the GeliS study was the proportion of pregnant women who showed an excessive weight gain during pregnancy according to the definition of the NAM [5]. Both in the intervention and control groups, the proportion of women with excessive GWG was around 45% [21]. Data on the intervention effects of other maternal and infant health outcomes have already been published [24–29]. At study entry women reported anthropometric and sociodemographic characteristics, information about their country of birth and number of previous births in a screening questionnaire. Pre-pregnancy BMI category was calculated from self-reported pre-pregnancy weight and height. Data on pre-pregnancy and early pregnancy lifestyle, such as dietary and physical activity behaviours, mental health status, and smoking behaviour were collected via a questionnaire set before the 12^th^ week of gestation. Dietary and physical activity behaviour were evaluated with a validated Food Frequency Questionnaire and the Pregnancy Physical Activity Questionnaire, respectively [30, 31]. Details on the assessment and evaluation of the dietary and physical activity behaviours have already been described in detail [24, 25]. Mental wellbeing was assessed by means of the World Health Organization Well-Being Index (WHO-5) [32]. Signs of depressive disorder were identified using the first two questions of the Patient Health Questionnaire-4 (PHQ-4), a ultra-short screening scale for anxiety and depression [33]. A PHQ-2 score of at least three points indicated depressive symptoms [34]. Women´s weight was continuously measured throughout pregnancy and entered in maternity records as part of routine care visits. GWG was calculated as the difference between the maternal weight measured at the last prenatal visit and the weight measured at the first routine prenatal visit before the 12^th^ week of gestation.

### The FeLIPO study

The FeLIPO (“Feasibility of lifestyle-intervention in pregnancy to optimize maternal weight development”) study was a cluster-randomised intervention trial that has been conducted as a pilot study for the GeliS trial [35]. In the present analysis, the FeLIPO data were used for external validation (see Statistical analyses). The primary endpoint of the FeLIPO study was the proportion of women showing an excessive GWG according to the NAM recommendations [5]. Between February 2010 and August 2011, 250 healthy pregnant women were recruited before the 18^th^ week of gestation in eight gynaecological practices in Munich, Germany. The inclusion and exclusion criteria were similar to those of the GeliS study. The FeLIPO lifestyle intervention consisted of two counselling sessions during pregnancy on the topics of healthy lifestyle and weight monitoring provided by a trained dietician. The intervention led to a lower proportion of women with excessive GWG in the intervention group compared to the control group (38% vs. 60%; *p* < 0.032). A detailed description of the FeLIPO study and its results can be found elsewhere [35, 36].

Definitions and measurements of anthropometric, sociodemographic, lifestyle and smoking data were similar to those in the GeliS dataset. No data were collected on the mental health of women. Other than previously published [35], small adjustments had to be made to the FeLIPO-variables to align them with the GeliS-variables: The FeLIPO categories of educational level were reduced from four to three categories by combining the categories high school/grammar school and university degree from the raw dataset. The FeLIPO variable GWG was re-calculated and instead of the self-reported weight before pregnancy, women´s measured weight at study entry and at the last prenatal visit before delivery was used to determine GWG.

### Statistical analyses

For this analysis, all GeliS participants with a full-term delivery as well as no missing data for GWG and all included variables were considered. Those women who dropped out during the intervention phase of the study were excluded. As no difference in GWG was seen between the two groups, women in the intervention and control groups were pooled to form one cohort. In all analyses, group assignment was included as a covariate. Maternal baseline characteristics are presented with mean ± standard deviation (SD) for continuous variables and with numbers and proportions for categorical variables. The descriptive statistics were stratified according to whether excessive GWG occurred or not. Statistical differences between women with and without excessive GWG were examined using the χ2 test for categorical variables and the Kruskal–Wallis test for continuous variables.

#### Risk factors

Potential risk factors, collected before the 12^th^ week of gestation, were determined a priori to develop the risk score for excessive GWG. The selection of variables was based on the evidence from previous literature regarding the hypothesised association of sociodemographic and anthropometric factors, smoking behaviour and mental health status of women and their potential influence on GWG [12–20]. The following categorical variables were included in the development of the risk score model:

- pre-pregnancy BMI category (BMI 18.5–24.9 kg/m^2^ vs. BMI 25.0–29.9 kg/m^2^ vs. BMI 30.0–40.0 kg/m^2^)
- pre-pregnancy age (18–25 years vs. 26–35 years vs. 36–43 years)
- educational level (not (yet) graduated from school/general secondary school vs. intermediate secondary school vs. high school)
- country of birth (Germany vs. foreign country)
- parity (nulliparous vs. multiparous)
- smoking status (never smoker vs. current and/or former smoker)
- signs of depressive disorder (PHQ-2 score ≥ 3 vs. PHQ-2 score < 3)
- full-time employed (yes vs. no)

#### Risk score model development

The GeliS cohort was randomly split into two subsets: the development dataset and the validation dataset with a ratio of 80:20. The development set (80% of GeliS participants) was used to create the screening questionnaire based on a risk score. Therefore, a binary multivariate logistic regression analysis with stepwise backward elimination was performed to obtain odds ratios (OR) and 95% confidence intervals (CI) for a reduced set of variables that best predicted the occurrence of excessive GWG. The stepwise backward elimination started with all potential risk factors mentioned above. The Akaike information criterion was used to create the best-balanced model to evade under- and overfitting. The scoring system for our screening questionnaire was based on the methodological approach of Sullivan et al. [37]. Each β coefficient of the final variables was divided by the lowest β coefficient of the model and rounded to the nearest integer to obtain individual points. The total risk score was calculated as the sum of the individual points indicating a higher risk with a higher score value.

#### Risk score model validation

To evaluate the performance of the risk score model, the score was cross validated using the validation dataset (20% of GeliS participants), and additionally externally validated within the FeLIPO pooled cohort. Data on the independent variables were available from all 250 participants of the FeLIPO trial and included for the external validation analysis, even though data on the dependent variable GWG were missing from 25 participants. The receiver operating characteristic (ROC) curves were built by plotting the sensitivity on the y-axis and the false positive rate (1-specificity) on the x-axis based on logistic regression models. The area under the curve (AUC) was used to assess the model in terms of discrimination (the ability to differ between individuals who experienced excessive GWG and those who did not).

Statistical analysis was performed with RStudio software (RStudio Inc., version 4.0.3, Boston, MA, USA) and SPSS software (IBM SPSS Statistics for Windows, version 26.0, IBM Corp, Armonk, NY, USA). A significance level of 5% was considered statistically significant. No adjustment for multiple testing was performed due to the exploratory approach of the analysis.

## Results

### Flowchart and baseline characteristics

**Fig. 1:**
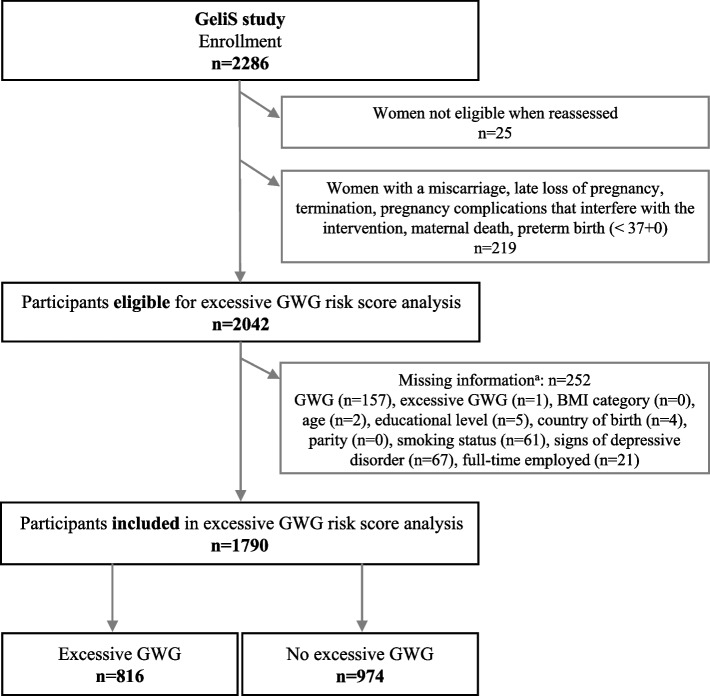
Flowchart of GeliS participants considered for excessive GWG risk score analysis. Legend: Abbreviation: GeliS: Gesund leben in der Schwangerschaft (healthy living in pregnancy); GWG: Gestational weight gain; BMI: Body mass index. ^a^ multiple reasons possible

Of the 2286 women initially recruited for the GeliS study, 2042 women were potentially eligible for the analysis (Fig. 1). Due to missing data in the potential risk factors, 252 women had to be excluded. In total, 1790 women were included in the risk score analysis of which 816 women showed excessive weight gain during pregnancy.

Table 1 presents maternal anthropometric, sociodemographic and lifestyle characteristics categorised by the occurrence of excessive GWG. 45.6% of women experienced excessive GWG. Among these women, the average age was lower (29.8 ± 4.2 years vs. 30.6 ± 4.5 years, *p* < 0.001), and the average self-reported weight before pregnancy was higher (71.8 ± 13.2 kg vs. 65.1 ± 12.6 kg, *p* < 0.001) compared to women without excessive GWG (54.4%). The between-group differences in pre-pregnancy BMI category and educational level were statistically significant (*p* < 0.001, respectively). About one in ten women in both groups indicated a country outside Germany as their country of birth. A higher rate of women with excessive GWG was nulliparous (63.5% vs. 52.7%, *p* < 0.001), full-time employed (58.1% vs. 48.9%, *p* < 0.001), and indicated to be a former or current smoker (56.6% vs. 41.7%, *p* < 0.001). Participants who experienced excessive GWG were less likely to meet the general recommendations for physical activity (56.1% vs. 51.0%, *p* = 0.034) and showed lower well-being during pregnancy (38.8% vs. 34.4%, *p* = 0.052) compared to participants with adequate weight gain. No significant differences between the groups were observed regarding diet quality (26.2% vs. 23.9%, *p* = 0.267) and signs of anxiety and depression (44.4% vs. 40.3%, *p* = 0.078).

**Table 1:** Baseline characteristics of GeliS participants in relation to excessive GWG

	**Excessive** **GWG** (*n* = 816, 45.6%)	**No excessive** **GWG** (*n* = 974, 54.4%)	**Total** (*n* = 1790)	***p* value**^**a**^
Group allocation, n (%)^b^				0.762
Control group	408/816 (50.0%)	480/974 (49.3%)	888/1790 (49.6%)	
Intervention group	408/816 (50.0%)	494/974 (50.7%)	902/1790 (50.4%)	
Pre-pregnancy age (years)^c^	29.8 ± 4.2	30.6 ± 4.5	30.3 ± 4.4	< 0.001
Pre-pregnancy weight (kg)	71.8 ± 13.2	65.1 ± 12.6	68.2 ± 13.3	< 0.001
Pre-pregnancy BMI (kg/m^2^)	25.4 ± 4.4	23.4 ± 4.2	24.3 ± 4.4	< 0.001
Pre-pregnancy BMI category				< 0.001
BMI 18.5–24.9 kg/m^2^	414/816 (50.7%)	760/974 (78.0%)	1174/1790 (65.6%)	
BMI 25.0–29.9 kg/m^2^	274/816 (33.6%)	132/974 (13.6%)	406/1790 (22.7%)	
BMI 30.0–40.0 kg/m^2^	128/816 (15.7%)	82/974 (8.4%)	210/1790 (11.7%)	
Educational level ^d^				< 0.001
General secondary school	143/816 (17.5%)	132/974 (13.6%)	275/1790 (15.4%)	
Intermediate secondary school	379/816 (46.4%)	389/974 (39.9%)	768/1790 (42.9%)	
High school	294/816 (36.0%)	453/974 (46.5%)	747/1790 (41.7%)	
Country of birth				0.279
Germany	720/816 (88.2%)	875/974 (89.8%)	1595/1790 (89.1%)	
Others	96/816 (11.8%)	99/974 (10.2%)	195/1790 (10.9%)	
Nulliparous	518/816 (63.5%)	513/974 (52.7%)	1031/1790 (57.6%)	< 0.001
Living with a partner	790/814 (97.1%)	936/971 (96.4%)	1726/1785 (96.7%)	0.440
Full-time employed	474/816 (58.1%)	476/974 (48.9%)	950/1790 (53.1%)	< 0.001
Current or former smoker	462/816 (56.6%)	406/974 (41.7%)	868/1790 (48.5%)	< 0.001
Low diet quality ^e^	208/793 (26.2%)	227/949 (23.9%)	435/1742 (25.0%)	0.267
Low physical activity ^f^	434/774 (56.1%)	481/944 (51.0%)	915/1718 (53.3%)	0.034
Low wellbeing ^g^	312/805 (38.8%)	331/965 (34.4%)	643/1770 (36.3%)	0.052
Signs of anxiety and depression ^h^	361/813 (44.4%)	391/971 (40.3%)	752/1784 (42.2%)	0.078

*Abbreviation*: *GeliS* Gesund leben in der Schwangerschaft (healthy living in pregnancy), *GWG* Gestational weight gain, defined by the NAM criteria [5], *BMI* Body mass index, *SD* Standard deviation, *MET* Metabolic equivalent of task

^a^*p* value for differences between women with and without excessive GWG

^b^Frequency (percent) (all such values)

^c^Mean ± SD (all such values)

^d^General secondary school: General school, which is completed through year 9; Intermediate secondary school: Vocational secondary school, which is completed through year 10; High school: Academic high school, which is completed through year 12 or 13

^e^Low diet quality determined by means of the Healthy Eating Index [24] below the 25^th^ quartiles

^f^Not meeting physical activity recommendation defined as ≤ 7.5MET-h/week in category sports activity of moderate-intensity or greater [25] determined by means of the Pregnancy Physical Activity Questionnaire [31]

^g^Low wellbeing defined by means of the World Health Organization Well-Being Index < 50 [32]

^h^Anxiety and depression are assessed by means of a Patient Health Questionnaire-4 score of ≥ 3 points [33]

### Development of the screening questionnaire

A total of 1432 (80%) and 358 (20%) women were included in the development and validation cohorts, respectively. Maternal characteristics in each cohort were comparable (Additional file 1: Table S1). The stepwise backward elimination resulted in six risk factors that retained in the model as variables that best predicted the occurrence of excessive GWG: pre-pregnancy BMI category, educational level, country of birth, parity, smoking status, and signs of depressive disorder. Additional file 2 (Table S2) shows the multivariate logistic regression of the considered potential risk factors prior to the stepwise backward elimination. Maternal age and full-time employment as the variables with the smallest partial correlation to the dependent variable of excessive GWG were removed by the stepwise backward elimination approach. Table 2 shows results of the final variables, their β coefficients, OR (95% CI) and *p* values, that remained in the model and were associated with the risk for excessive GWG.

**Table 2:** Multivariate regression model of maternal characteristics predicting excessive GWG, after stepwise backward elimination (*n* = 1432)

	**β coefficient**	**OR (95% CI)**^**a**^	***p* value**^**a**^	**Points allocated**^**b**^
**Pre-pregnancy BMI category**				
Normal weight	Reference			0
Overweight	1.43	4.17 (3.16–5.53)	< 0.001	7
Obesity	0.94	2.55 (1.82–3.60)	< 0.001	4
**Educational level**^**c**^				
General secondary school	0.21	1.23 (0.87–1.73)	0.234	1
Intermediate secondary school	0.28	1.32 (1.03–1.68)	0.026	1
High school	Reference			0
**Country of birth**				
Germany	Reference			0
Others	0.31	1.36 (0.96–1.93)	0.085	1
**Nulliparity**				
No	Reference			0
Yes	0.60	1.82 (1.45–2.30)	< 0.001	3
**Ever smoked**				
No	Reference			0
Yes	0.39	1.47 (1.17–1.85)	< 0.001	2
**Signs of depressive disorder**^**d**^				
No				0
Yes	0.28	1.32 (0.94–1.86)	0.114	1

*Abbreviation*: *GWG* Gestational weight gain, *OR* Odds Ratio, *CI* Confidence Interval, *BMI* Body mass index

^a^Adjusted for group assignment

^b^Allocated points: each β coefficient is divided by the lowest β coefficient and rounded to the nearest integer

^c^General secondary school: General school, which is completed through year 9; Intermediate secondary school: Vocational secondary school, which is completed through year 10; High school: Academic high school, which is completed through year 12 or 13

^d^Signs of depressive disorder is assessed by means of a Patient Health Questionnaire-2 score of ≥ 3 points [34]

The β coefficients of each factor were divided by 0.21 as the lowest β coefficient of the model and then rounded to the nearest integer to obtain individual points (last column of Table 2). The reference categories were given a score of 0, the total score as the sum of the individual points ranged from 0 to 15 (Table 2). The risk was classified into low (0–5), moderate (6–10) and high (11–15) risk according to the total score. A German and English version of the practical screening questionnaire and its point allocation scheme is presented in Additional file 3 (Table S3-S4) and Additional file 4 (Table S5-S6), respectively.

### Validation of the screening questionnaire

**Fig. 2:**
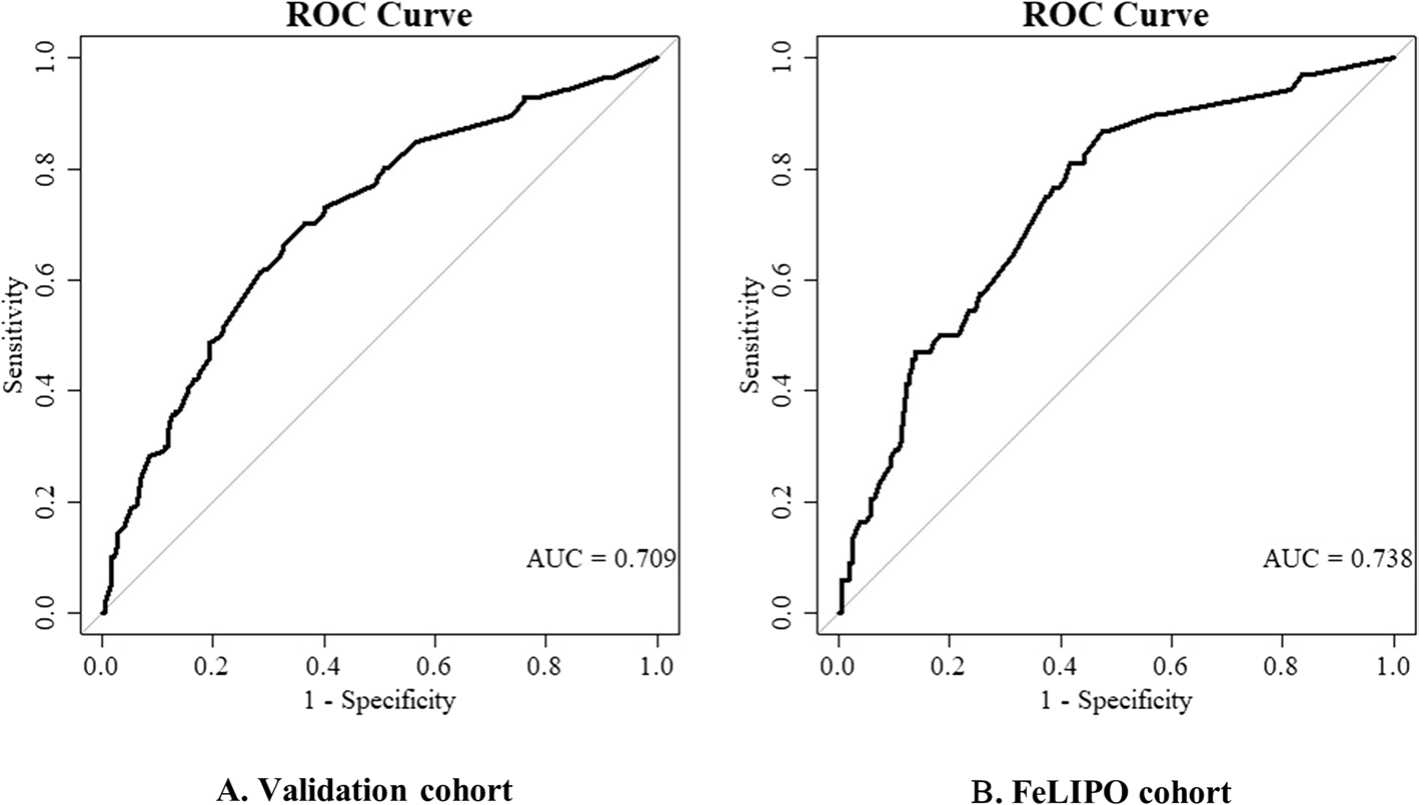
The area under the receiver operating characteristic curve for the screening questionnaire of excessive GWG. Legend: **A** ROC curve in the GeliS validation cohort (*n* = 358). **B** ROC curve in the external FeLIPO cohort (*n* = 250). Abbreviation: GWG: Gestational weight gain; ROC: Receiver operating characteristic; AUC: Area under curve; GeliS: Gesund leben in der Schwangerschaft (healthy living in pregnancy); FeLIPO: Feasibility of lifestyle-intervention in pregnancy to optimize maternal weight development. The diagonal lines are the reference lines (random classification)

Figure 2A shows the result of the ROC curve in the validation dataset (20%, *n* = 358). The model yielded an AUC of 0.709 (95% CI 0.66–0.76) indicating a correct discrimination of 70.9% of women with and without excessive GWG. Figure 2B presents the results of the external validation of the risk score model by using the independent FeLIPO pilot study sample (*n* = 250). The discriminatory power assessed by the AUC was 0.738 (95% CI 0.67–0.81).

## Discussion

Using pooled data from the large-scale GeliS trial, a screening tool for early identification of women at risk for excessive GWG was developed and validated. By means of a multivariate logistic regression analysis with stepwise backward elimination, we identified six variables as risk factors for excessive GWG: higher maternal pre-pregnancy BMI, intermediate educational level, nulliparity, being born in a country outside Germany, having ever smoked, and signs of depressive disorder. All variables have already been associated with weight gain in pregnancy in systematic reviews and meta-analyses of the current literature [12–14, 19, 20, 38]. So far, the combined consideration of potential risk factors for excessive GWG within one model, and thereupon the development and validation of an applicable screening tool, has not yet been undertaken. The ROC statistics of the final variables resulted in an AUC of 0.709 within the validation dataset and an AUC of 0.738 in the external FeLIPO dataset. These results indicate that 70.9% and 73.8% of women can correctly be classified, respectively, which suggests a moderate discriminatory power [39, 40]. The scoring system of our screening questionnaire provides the classification into different risk categories according to women’s estimated risk of excessive GWG.

So far, research on developing screening tools has been adopted in other populations and contexts, for example the prevention of diabetes mellitus [41, 42] or GDM [43–45]. In an external validation study by Lamain-de Ruiter et al. of twelve published models for predicting GDM, the ROC statistic of included studies ranged from 0.67 to 0.78 [46], which is comparable to our results. Previous screening tools during pregnancy intended to predict either a high risk for maternal vitamin D deficiency [47] or for caesarean delivery among women with GDM [48]. With respect to excessive GWG, only one study to date has examined nine psychological, physical, and social factors as risk factors for excessive GWG within a cross-validated prediction model in 970 women (AUC 0.62) [49], but without validating the results against an external study population or implementing them as part of a screening tool. Another study from Iceland developed a dietary screening questionnaire using stepwise backward elimination to predict excessive GWG, but no validation was performed [50].

A variety of factors should be considered when examining determinants of excessive GWG, as weight gain is a composite outcome resulting from a multi-faceted interaction between genetics [51], physiological and metabolic processes, placental metabolism, and other factors including environmental [51], personal, behavioural [5, 12] and psychological [38]. Our screening questionnaire is based exclusively on maternal risk factors derived from early pregnancy data. This allows the identification of vulnerable individuals for which targeted, and risk-adapted intervention strategies can be initiated at an early stage. Our findings detect pre-pregnancy overweight and obesity as strongest risk factors for excessive GWG, which is in line with current literature [12, 14, 52]. In Germany, the number of pregnant women with overweight and obesity has increased in recent years and currently almost 45% of pregnant women have a BMI greater than 24.9 kg/m^2^ at the beginning of their pregnancy [53]. Furthermore, our results have shown that nulliparous women were more likely to develop excessive GWG that has recently been confirmed in a comprehensive meta-analysis [12]. Multipara women, on the contrary, may have already experienced knowledge about prenatal health care and adequate lifestyle during pregnancy due to previous pregnancies. The present analysis observed that compared to women with a high school degree, women with an intermediate level of education showed an increased risk for excessive GWG, but not those with the lowest educational level. This could be due to a smaller sample size in the lowest educational category. Current literature discusses the question of whether GWG guidelines should be culturally adapted [54]. Compatible with previous research is our observation that being born in a foreign country increased the risk of excessive GWG [18]. However, it should be noted that 67% of our participants who were born in a foreign country indicated Russia, Kazakhstan, Poland, or Romania and therefore share a similar cultural background (data not shown). This might suggest that culture, ethnicity, or genetic resemblance may have influenced our results and impede comparability.

Maternal current or former smoking increases the risk for gaining gestational weight above recommendations, and presumably indicates a general unhealthier lifestyle. This theory is strengthened by the results of a study by Dallongeville et al. [55], which showed that smoking is related to higher intakes of total energy, total fat, and dietary saturated fat. Moreover, our findings show that excessive weight gain during pregnancy is favoured by the presence of depressive symptoms. The inclusion of the validated multi-brief PHQ-2 as a first step approach to determine depressive symptoms is a particular strength of our screening questionnaire, even though data on women´s mental health were not available in the FeLIPO dataset and thus could not be considered for external validation. The relationship between weight and depression disorder has already been shown previously both in the general population [56], but also among pregnant women [16]. There is increasing evidence that psychological factors during pregnancy are associated with excessive GWG, as they might play a crucial role in adopting healthy antenatal lifestyle habits for the purpose of weight management [16]. Therefore, early assessment and subsequent addressing of women´s mental health should be considered in the prevention of overweight and obesity.

Our screening questionnaire can identify women at risk even prior to conception, since the variables included in the screening tool are based on data from early pregnancy, but already relate to the time before pregnancy. This is a particular value since recent research increasingly emphasises the relevance of women’s preconception health in terms of weight management and healthy lifestyle, which should be given greater attention in both intervention approaches and clinical practice [57, 58]. A screening questionnaire for excessive GWG implemented before pregnancy also addresses the fact that many pregnancies are unintended [59].

In terms of practical implications, our screening questionnaire might be an assistive tool for future digital intervention trials. Furthermore, it addresses the current call for translating research findings into clinical practice in the understudied group of pregnant women [60]. As resource and time saving tool it could be used to scale recommendations for appropriate GWG into routine gynaecological care. This, in turn, might lead to a higher awareness on potential consequences of excessive GWG and might allow suitable corrective measures for women at risk, while avoiding spending resources for women without risk.

### Strengths and limitations

A strength of this study is the extensive GeliS dataset, which allowed us to consider the risk value of diverse candidate variables to be tested and included in the screening questionnaire. Further, the final variables are easily attainable from self-reported data, do not require clinical measurements, and could be included on a medical history form that women fill out at their gynaecologist practice. The large sample size and the low rate of missing data enabled both the development and validation of the screening questionnaire by splitting the dataset. Moreover, few research groups have validated their screening tools within an external study population, which is a particular strength of our approach.

The findings should be considered with respect to limitations. Some of the included variables, such as pre-pregnancy BMI and smoking status are susceptible to reporting bias, which may have resulted in an over- or underestimation of the reported impact on excessive GWG. Another limitation is the information on country of birth as it does not specify migration background or ethnicity. Furthermore, the assessment of excessive GWG according to NAM recommendations in the German population is disputed [61]. Nevertheless, the NAM criteria have been considered as a basis for assessing GWG in the GeliS cohort since they are generally applied in clinical trials and settings where clearly defined and measurable endpoints are required. NAM thresholds exist for each BMI category, which coincided with the aim of the GeliS study to include women from different BMI categories. They represent the current global standard [12]. Thus, the GeliS data correspond to the international literature, which enables comparability. Notwithstanding, the NAM guidelines as well as our screening questionnaires should always be used in concert with good clinical judgement of their practical application. Moreover, we are aware that the study samples used for the development and validation of our screening tool do not include high-risk pregnancies due to exclusion criteria, and participants were overall well-educated and from only one federal state in Germany. This limits the applicability of our findings to broader populations. Nevertheless, the occurrence of excessive GWG in the GeliS cohort was comparable with other population-based data [8, 62]. Further validations in national and international samples as well as testing in non-randomised settings are necessary for improving the external generalisability and investigating the clinical value of our findings.

## Conclusions

We have developed a new, validated screening questionnaire that is able to identify women at an early stage who are at risk for excessive GWG by obtaining self-reported data on maternal sociodemographics, anthropometrics, smoking behaviour, and mental health status. Our screening questionnaire can be used for tailored intervention strategies aimed at preventing excessive GWG and associated adverse pregnancy outcomes and could contribute to cost-efficiency in primary health care settings. Future studies should evaluate the applicability for routine use in clinical practice as well as the clinical benefit in terms of patient outcomes.

## Supplementary Information

**Additional file 1:** **Table S1.** Baseline characteristics of GeliS participants in the development and validation cohorts.

**Additional file 2:** **Table S2.** Multivariate regression model of potential maternal characteristics predicting excessive GWG, before stepwise backward elimination (*n*=1432).

**Additional file 3:** **Table S3.** Practical screening questionnaire (German version). **Table S4.** Point allocation scheme (German version).

**Additional file 4:** **Table S5.** Practical screening qestionnaire (English version). **Table S6.** Point allocation scheme (English version).

## Data Availability

The datasets used and analysed during the current study are available from the corresponding author on reasonable request.

## Abbreviations

AUC: Area under the curve
AUC ROC: Area under the receiver operating characteristic curve
BMI: Body mass index
CI: Confidence interval
FeLIPO: Feasibility of lifestyle-intervention in pregnancy to optimize maternal weight development
GDM: Gestational diabetes mellitus
GeliS: “Gesund leben in der Schwangerschaft”/ “healthy living in pregnancy”
GWG: Gestational weight gain
MET: Metabolic equivalent of task
NAM: National academy of medicine
OR: Odds ratio
PHQ: Patient health questionnaire
ROC: Receiver operating characteristics
SD: Standard deviation
TRIPOD: Transparent Reporting of a Multivariable Prediction Model for Individual Prognosis or Diagnosis
WHO-5: World Health Organization Well-Being Index-5
