# Supplementary material for: Development and validation of a screening questionnaire for early identification of pregnant women at risk for excessive gestational weight gain
Source: BMC Pregnancy Childbirth. 2023 Apr 13;23:249. doi: 10.1186/s12884-023-05569-7 (PMC10100402; doi:10.1186/s12884-023-05569-7)
Supplement: Supplementary file 1 — Additional file 1: Table S1. Baseline characteristics of GeliS participants in the development and validation cohorts. [file 12884_2023_5569_MOESM1_ESM.docx]

**Table S1:** Baseline characteristics of GeliS participants in the development and validation cohorts.

|  | **Development cohort**  (*n*=1432, 80.0%) | **Validation cohort** (*n*=358, 20.0%) | ***p* value^a^** |
| --- | --- | --- | --- |
| Excessive GWG, n (%)^b^ | 645/1432 (45.0%) | 171/358 (47.8%) | 0.400 |
| Group allocation |  |  | 0.300 |
| Control group | 702/1432 (49.0%) | 186/358 (52.0%) |  |
| Intervention group | 730/1432 (51.0%) | 172/358 (48.0%) |  |
| Pre-pregnancy age (years)^c^ | 30.3 (4.4) | 30.2 (4.5) | 0.700 |
| Pre-pregnancy weight (kg) | 68.3 (13.5) | 67.5 (12.5) | 0.500 |
| Pre-pregnancy BMI (kg/m^2^) | 24.4 (4.5) | 24.0 (4.2) | 0.300 |
| Pre-pregnancy BMI category |  |  | 0.500 |
| BMI 18.5–24.9 kg/m^2^ | 938/1432 (65.5%) | 236/358 (65.9%) |  |
| BMI 25.0–29.9 kg/m^2^ | 320/1432 (22.3%) | 86/358 (24.0%) |  |
| BMI 30.0–40.0 kg/m^2^ | 174/1432 (12.2%) | 36/358 (10.1%) |  |
| Educational level^d^ |  |  | 0.200 |
| General secondary school | 216/1432 (15.1%) | 59/358 (16.5%) |  |
| Intermediate secondary school | 603/1432 (42.1%) | 165/358 (46.1%) |  |
| High school | 613/1432 (42.8%) | 134/358 (37.4%) |  |
| Country of birth |  |  | 0.300 |
| Germany | 1.270/1432 (88.7%) | 325/358 (90.8%) |  |
| Others | 162/1432 (11.3%) | 33/358 (9.2%) |  |
| Nulliparous | 832/1432 (58.1%) | 199/358 (55.6%) | 0.900 |
| Living with a partner | 1.382/1428 (96.8%) | 344/357 (96.4%) | 0.700 |
| Full-time employed | 764/1432 (53.4%) | 186/358 (52.0%) | 0.600 |
| Current or former smoker | 681/1432 (47.6%) | 187/358 (52.2%) | 0.110 |
| Low diet quality^e^ | 336/1395 (24.1%) | 99/347 (28.5%) | 0.087 |
| Low physical activity^f^ | 723/1378 (52.5%) | 192/340 (56.5) | 0.200 |
| Low wellbeing^g^ | 504/1413 (35.7%) | 139/357 (38.9%) | 0.300 |
| Signs of anxiety and depression^h^ | 601/1427 (42.1%) | 151/357 (42.3%) | 0.900 |

Abbreviations: GeliS: Gesund leben in der Schwangerschaft (Healthy living in pregnancy); GWG: Gestational weight gain, defined by the NAM criteria (1) BMI: Body mass index; SD: Standard deviation; MET: Metabolic equivalent of task.

^a^ *p* value for differences between the women of the development and validation dataset (calculated with RStudio applying the best fitting model: Pearson´s Chi-squared test, Wilcoxon rank sum test, Fisher´s exact test).

^b^ Frequency (percent) (all such values).

^c^ Mean ± SD (all such values).

^d^ General secondary school: General school, which is completed through year 9; Intermediate secondary school: Vocational secondary school, which is completed through year 10; High school: Academic high school, which is completed through year 12 or 13.

^e^ Low diet quality determined by means of the Healthy Eating Index (2) below the 25^th^ quartiles.

^f^ Not meeting physical activity recommendation defined as ≤ 7.5 MET-h/week in category sports activity of moderate-intensity or greater (3) determined by means of the Pregnancy Physical Activity Questionnaire (4).

^g^ Low wellbeing defined by means of the World Health Organization Well-Being Index < 50 (5).

^h^ Anxiety and depression are assessed by means of a Patient Health Questionnaire-4 score of ≥ 3 points (6).

**References**

1. Rasmussen KM, Yaktine AL, editors. Weight Gain During Pregnancy: Reexamining the Guidelines. Washington, DC: National Academies Press; 2009.

2. Günther J, Hoffmann J, Kunath J, Spies M, Meyer D, Stecher L et al. Effects of a Lifestyle Intervention in Routine Care on Prenatal Dietary Behavior-Findings from the Cluster-Randomized GeliS Trial. J Clin Med 2019; 8(7).

3. Hoffmann J, Günther J, Geyer K, Stecher L, Rauh K, Kunath J et al. Effects of a lifestyle intervention in routine care on prenatal physical activity - findings from the cluster-randomised GeliS trial. BMC Pregnancy Childbirth 2019; 19(1):414.

4. Chasan-Taber L, Schmidt MD, Roberts DE, Hosmer D, Markenson G, Freedson PS. Development and validation of a Pregnancy Physical Activity Questionnaire. Med Sci Sports Exerc 2004; 36(10):1750–60.

5. Topp CW, Østergaard SD, Søndergaard S, Bech P. The WHO-5 Well-Being Index: a systematic review of the literature. Psychother Psychosom 2015; 84(3):167–76.

6. Kroenke K, Spitzer RL, Williams JBW, Löwe B. An ultra-brief screening scale for anxiety and depression: the PHQ-4. Psychosomatics 2009; 50(6):613–21.
