# Supplementary material for: Development and validation of a screening questionnaire for early identification of pregnant women at risk for excessive gestational weight gain
Source: BMC Pregnancy Childbirth. 2023 Apr 13;23:249. doi: 10.1186/s12884-023-05569-7 (PMC10100402; doi:10.1186/s12884-023-05569-7)
Supplement: Supplementary file 2 — Additional file 2: Table S2. Multivariate regression model of potential maternal characteristics predicting excessive GWG, before stepwise backward elimination (n=1432). [file 12884_2023_5569_MOESM2_ESM.docx]

**Table S2:** Multivariate regression model of potential maternal characteristics predicting excessive GWG, before stepwise backward elimination (n=1432).

|  | **β coefficient** | **OR (95% CI)^a^** | ***p* value^a^** |
| --- | --- | --- | --- |
| **Pre-pregnancy BMI category** | | | |
| Normal weight | Reference | | |
| Overweight | 1.44 | 4.21 (3.19–5.60) | <0.001 |
| Obesity | 0.97 | 2.63 (1.88–3.72) | <0.001 |
| **Pre-pregnancy age** |  |  |  |
| 18–25 | 0.35 | 1.43 (0.90–2.27) | 0.132 |
| 26–35 | 0.36 | 1.43 (1.01–2.04) | 0.049 |
| 36–43 | Reference | | |
| **Educational level^b^** |  | | |
| General secondary school | 0.21 | 1.24 (0.87–1.75) | 0.230 |
| Intermediate secondary school | 0.26 | 1.29 (1.01–1.66) | 0.042 |
| High school | Reference | | |
| **Country of birth** | | | |
| Germany | Reference | | |
| Others | 0.30 | 1.35 (0.95–1.92) | 0.092 |
| **Nulliparity** | | | |
| No | Reference | | |
| Yes | 0.43 | 1.54 (1.12–2.11) | 0.008 |
| **Ever smoked** |  | | |
| No | Reference | | |
| Yes | 0.39 | 1.48 (1.18–1.86) | <0.001 |
| **Full-time employed** |  |  |  |
| No | Reference | | |
| Yes | 0.21 | 1.23 (0.91–1.67) | 0.179 |
| **Signs of depressive disorder^c^** |  |  |  |
| No | Reference | | |
| Yes | 0.28 | 1.32 (0.93–1.86) | 0.116 |

Abbreviation: GWG: Gestational weight gain; OR: Odds Ratio; CI: Confidence Interval; BMI: Body mass index.

^a^ Adjusted for group assignment.

^b^ General secondary school: General school, which is completed through year 9; Intermediate secondary school: Vocational secondary school, which is completed through year 10; High school: Academic high school, which is completed through year 12 or 13.

^c^ Signs of depressive disorder are assessed by means of a Patient Health Questionnaire-2 score of ≥ 3 points (1).

**References**

1. Kroenke K, Spitzer RL, Williams JBW. The Patient Health Questionnaire-2: validity of a two-item depression screener. Med Care 2003; 41(11):1284–92.
