# Supplementary material for: Development and validation of a screening questionnaire for early identification of pregnant women at risk for excessive gestational weight gain
Source: BMC Pregnancy Childbirth. 2023 Apr 13;23:249. doi: 10.1186/s12884-023-05569-7 (PMC10100402; doi:10.1186/s12884-023-05569-7)
Supplement: Supplementary file 3 — Additional file 3: Table S3. Practical screening questionnaire (German version). Table S4. Point allocation scheme (German version). [file 12884_2023_5569_MOESM3_ESM.docx]

**Table S3:** Practical screening questionnaire (German version).

**Bitte beantworten Sie die nachfolgenden Fragen.** Die Einzelpunkte und der Gesamt-Score werden von Ihrer Ärztin/Ihrem Arzt bestimmt. Der Gesamt-Score beschreibt Ihr Risiko für eine exzessive Gewichtszunahme in der Schwangerschaft.

| **Ihr Körpergewicht vor Schwangerschaftsbeginn:** ____, __ kg |  |
| --- | --- |
| **Ihre Köpergröße:** _______ cm |  |
| **Was ist Ihr höchster Schulabschluss?** |  |
| (Noch) keinen Schulabschluss |  |
| Hauptschule/Volksschule |  |
| Mittlere Reife/Realschule |  |
| Fachhochschulreife/Abitur |  |
| **Ihr Geburtsland:** |  |
| Deutschland |  |
| Anderes Geburtsland |  |
| **Haben Sie bereits Kinder geboren?** |  |
| Nein |  |
| Ja, ich habe bereits Kinder geboren. |  |
| **Haben Sie jemals regelmäßig geraucht?** |  |
| Nein |  |
| Ja |  |
| **Wie oft fühlten Sie sich im Verlauf der letzten 2 Wochen durch die folgenden Beschwerden beeinträchtigt?** | |
| **…wenig Interesse oder Freude an Ihren Tätigkeiten** |  |
| Überhaupt nicht |  |
| An einzelnen Tagen |  |
| An mehr als die Hälfte der Tage |  |
| Beinahe jeden Tag |  |
| **…Niedergeschlagenheit, Schwermut, Hoffnungslosigkeit** |  |
| Überhaupt nicht |  |
| An einzelnen Tagen |  |
| An mehr als die Hälfte der Tage |  |
| Beinahe jeden Tag |  |

|  | | |  | **Einzelpunkte** |
| --- | --- | --- | --- | --- |
| **Body Maß Index:** | | |  |  |
| Untergewicht (< 18,5 kg/m^2^) | | |  | **0** |
| Normalgewicht (18,5–24,9 kg/m^2^) | | |  | **0** |
| Übergewicht (25,0–29,9 kg/m^2^) | | |  | **7** |
| Adipositas (> 30,0 kg/m^2^) | | |  | **4** |
| **Schulabschluss:** | | |  |  |
| (Noch) keinen Schulabschluss | | |  | **1** |
| Hauptschule/Volksschule | | |  | **1** |
| Mittlere Reife/Realschule | | |  | **1** |
| Fachhochschulreife/Abitur | | |  | **0** |
| **Geburtsland:** | | |  |  |
| Deutschland | | |  | **0** |
| Anderes Geburtsland | | |  | **1** |
| **Haben Sie bereits Kinder geboren?** | | |  |  |
| Ja, ich habe bereits Kinder geboren. | | |  | **0** |
| Nein | | |  | **3** |
| **Haben Sie jemals regelmäßig geraucht?** | | |  |  |
| Nein | | |  | **0** |
| Ja | | |  | **2** |
| **Wie oft fühlten Sie sich im Verlauf der letzten 2 Wochen durch die folgenden Beschwerden beeinträchtigt?** | | | | |
| **Wenig Interesse oder Freude an Ihren Tätigkeiten** | | |  |  |
| Überhaupt nicht | | | 0 |  |
| An einzelnen Tagen | | | 1 |  |
| An mehr als die Hälfte der Tage | | | 2 |  |
| Beinahe jeden Tag | | | 3 |  |
| **Niedergeschlagenheit, Schwermut, Hoffnungslosigkeit** | | |  |  |
| Überhaupt nicht | | | 0 |  |
| An einzelnen Tagen | | | 1 |  |
| An mehr als die Hälfte der Tage | | | 2 |  |
| Beinahe jeden Tag | | | 3 |  |
| Summe: | | | **____** |  |
|  | | | **≥ 3** | **1** |
|  | | | **< 3** | **0** |
| **Gesamt-Score:** | | | | __________ |
| Niedriges Risiko | Mittleres Risiko | Hohes Risiko | | |
| 0–5 | 6–10 | 11–15 | | |

**Table S4:** Point allocation scheme (German version).
