# Supplementary material for: Development and validation of a screening questionnaire for early identification of pregnant women at risk for excessive gestational weight gain
Source: BMC Pregnancy Childbirth. 2023 Apr 13;23:249. doi: 10.1186/s12884-023-05569-7 (PMC10100402; doi:10.1186/s12884-023-05569-7)
Supplement: Supplementary file 4 — Additional file 4: Table S5. Practical screening qestionnaire (English version). Table S6. Point allocation scheme (English version). [file 12884_2023_5569_MOESM4_ESM.docx]

**Table S5:** Practical screening qestionnaire (English version).

**Please answer the following questions.** The individual points and the total score are determined by your physician. The total score describes your risk for excessive gestational weight gain.

| **Your body weight before pregnancy:** ____, __ kg |  |
| --- | --- |
| **Your height:** _______ cm |  |
| **What is your highest school degree?** |  |
| Not (yet) graduated from school |  |
| General secondary school |  |
| Intermediate secondary school |  |
| High school |  |
| **Your country of birth:** |  |
| Germany |  |
| Other country of birth |  |
| **Have you already given birth?** |  |
| No |  |
| Yes, I have already born children. |  |
| **Did you ever smoke regularly?** |  |
| No |  |
| Yes |  |
| **Over the last 2 weeks, how often have you been bothered by the following problems?** | |
| **Little interest or pleasure in doing things** |  |
| Not at all |  |
| Several days |  |
| More than half the days |  |
| Nearly every day |  |
| **Feeling down, depressed, or hopeless** |  |
| Not at all |  |
| Several days |  |
| More than half the days |  |
| Nearly every day |  |

**Table S6:** Point allocation scheme (English version).

|  | | |  | **Individual points** |
| --- | --- | --- | --- | --- |
| **Body mass index:** | | |  |  |
| Underweight (< 18.5 kg/m^2^) | | |  | **0** |
| Normal weight (18.5–24.9 kg/m^2^) | | |  | **0** |
| Overweight (25.0–29.9 kg/m^2^) | | |  | **7** |
| Obesity (> 30.0 kg/m^2^) | | |  | **4** |
| **Educational level^a^:** | | |  |  |
| Not (yet) graduated from school | | |  | **1** |
| General secondary school | | |  | **1** |
| Intermediate secondary school | | |  | **1** |
| High school | | |  | **0** |
| **Country of birth:** | | |  |  |
| Germany | | |  | **0** |
| Other country of birth | | |  | **1** |
| **Have you already given birth?** | | |  |  |
| Yes, I have already born children. | | |  | **0** |
| No | | |  | **3** |
| **Did you ever smoke regularly?** | | |  |  |
| No | | |  | **0** |
| Yes | | |  | **2** |
| **Over the last 2 weeks, how often have you been bothered by the following problems?** | | | | |
| **Little interest or pleasure in doing things** | | |  |  |
| Not at all | | | 0 |  |
| Several days | | | 1 |  |
| More than half the days | | | 2 |  |
| Nearly every day | | | 3 |  |
| **Feeling down, depressed, or hopeless** | | |  |  |
| Not at all | | | 0 |  |
| Several days | | | 1 |  |
| More than half the days | | | 2 |  |
| Nearly every day | | | 3 |  |
| Sum: | | | **____** |  |
|  | | | **≥ 3** | **1** |
|  | | | **< 3** | **0** |
| **Total risk score:** | | | | __________ |
| Low risk | Moderate risk | High risk | | |
| 0–5 | 6–10 | 11–15 | | |

^a^ General secondary school: General school, which is completed through year 9; Intermediate secondary school: Vocational secondary school, which is completed through year 10; High school: Academic high school, which is completed through year 12 or 13.
